# College and Hospital Notes

**Published:** 1906-10

**Authors:** 


### COLLEGE AND HOSPITAL NOTES.

The trustees of the Buffalo General Hospital have purchased a plot of land on Goodrich Street which joins property now owned by the hospital, with the object of ultimately constructing thereon a children's hospital. Dr. D. W. Harrington provided a sum in his will for this purpose, the amount of the bequest not being known at present. It is thought, however, that it will yield between $75,000 and $100,000, which will amply provide for the ground and building. The purchase price of the ground just bought was $8,500, advantage being taken of this favorable opportunity to secure a site for the proposed children's hospital.

The Buffalo Woman's Hospital Training School for Nurses held its commencement exercises and reception at the Chapter House, Monday evening September 17, 1906, at which the following-named nurses were graduated: Elsie M. Carnes, Clara S. Lynn, Ruth G. Hall.

The following program was observed: Prayer, Rev. S. H. Coman; Address, Dr. Ida C. Bender; vocal selection, Mrs. A. H. Prentiss; announcements, Dr. C. C. Frederick; violin solo, Dr. J. A. Ragone; presentation of diplomas, Dr. Earl P. Lothrop. After the exercises were concluded there was dancing.

